# Molecular Dynamics of CH_4_/N_2_ Mixtures on a Flexible Graphene Layer: Adsorption and Selectivity Case Study

**DOI:** 10.3389/fchem.2019.00386

**Published:** 2019-06-03

**Authors:** Jelle Vekeman, Noelia Faginas-Lago, Andrea Lombardi, Alfredo Sánchez de Merás, Inmaculada García Cuesta, Marzio Rosi

**Affiliations:** ^1^Dipartimento di Chimica, Biologia e Biotecnologie, Università degli Studi di Perugia, Perugia, Italy; ^2^Instituto de Ciencia Molecular, Universidad de Valencia, Valencia, Spain; ^3^Consortium for Computational Molecular and Materials Sciences (CMS2), Perugia, Italy; ^4^Departamento de Química Física, Universidad de Valencia, Valencia, Spain; ^5^Dipartimento di Ingegneria Civile e Ambientale, Università degli Studi di Perugia, Perugia, Italy

**Keywords:** adsorption, molecular dynamics, *ab-initio* potential, flexible graphene, *ab initio* calculations

## Abstract

We theoretically investigate graphene layers, proposing them as membranes of subnanometer size suitable for CH_4_/N_2_ separation and gas uptake. The observed potential energy surfaces, representing the intermolecular interactions within the CH_4_/N_2_ gaseous mixtures and between these and the graphene layers, have been formulated by adopting the so-called Improved Lennard-Jones (ILJ) potential, which is far more accurate than the traditional Lennard-Jones potential. Previously derived ILJ force fields are used to perform extensive molecular dynamics simulations on graphene's ability to separate and adsorb the CH_4_/N_2_ mixture. Furthermore, the intramolecular interactions within graphene were explicitly considered since they are responsible for its flexibility and the consequent out-of-plane movements of the constituting carbon atoms. The effects on the adsorption capacity of graphene caused by introducing its flexibility in the simulations are assessed via comparison of different intramolecular force fields giving account of flexibility against a simplified less realistic model that considers graphene to be rigid. The accuracy of the potentials guarantees a quantitative description of the interactions and trustable results for the dynamics, as long as the appropriate set of intramolecular and intermolecular force fields is chosen. In particular it is shown that only if the flexibility of graphene is explicitly taken into account, a simple united-atom interaction potential can provide correct predictions. Conversely, when using an atomistic model, neglecting in the simulations the intrinsic flexibility of the graphene sheet has a minor effect. From a practical point of view, the global analysis of the whole set of results proves that these nanostructures are versatile materials competitive with other carbon-based adsorbing membranes suitable to cope with CH_4_ and N_2_ adsorption.

## 1. Introduction

Graphene has often been investigated as a possible material for the separation of small gases (Du et al., 2011; Wang et al., 2016; Raghavan and Gupta, 2017). Due to its unique structure and electronic properties on one hand and to its cheapness and stability on the other, it has been shown to outperform other candidate materials such as metal organic frameworks (MOF) in multiple studies (Thierfelder et al., 2011; Kumar et al., 2013; Wu et al., 2015). Usually, in molecular dynamics studies, the inherent internal flexibility of the graphene sheet is neglected by fixating the sheet within the simulation box. In reality, however, the sheet will have vibrations and other internal movement both in and out of the plane that might influence the adsorption capacity of the sheet (Deng and Berry, 2015; Bianco et al., 2018; Zhang et al., 2018). Indeed, in previous studies, we found that the adsorption of methane, nitrogen and carbon monoxide—separately simulated as pure gases adsorbing on graphene—was influenced by the introduction of a intramolecular force field in the graphene sheet in comparison to its rigid form (Vekeman et al., 2018b; Wilson et al., 2018; Vekeman et al., in press). In this work, we follow up on these previous works with a classical molecular dynamics study of the methane/nitrogen gas mixture in order to assess the influence of flexibility on the separation of both gases. In a similar way as in these previous works, we will use three intramolecular force fields —including stretch, bending and torsional terms—found in the literature that we have implemented and compare their performance to the typically modeled rigid sheet fixed in the simulation box (Walther et al., 2001; Kalosakas et al., 2013; Fthenakis et al., 2017).

Furthermore, we will evaluate the performance of different molecular gas models by comparing the estimates obtained from both united-atom and atomistic models for methane and nitrogen (Do and Do, 2005). The united-atom model, reducing the molecule to a sphere, is expected to be a cheap, yet reliable model, while the atomistic one, considering explicitly all interatomic interactions, will be more expensive and more accurate (Vela and Huarte-Larrañaga, 2011; Apriliyanto et al., 2018). The choice between these types of models is thus often a balancing of accuracy and computational cost and therefore their comparison is critical to make informed decisions (Lucena et al., 2010). In previous works, we have derived intramolecular potentials specifically designed for the adsorption of nitrogen and methane on graphene (Vekeman et al., 2018a,b). We based these potentials on the Improved Lennard-Jones potential (ILJ) (Pirani et al., 2004, 2008) as these have shown to outperform the very popular Lennard-Jones (LJ) potential (Pacifici et al., 2013; Faginas-Lago et al., 2015, 2016).

As the use of fossil fuels is causing more and more problems for the environment, a possible alternative on the short term is methane (Harfoot et al., 2018). It is more abundant on earth, cheaper and more easily implemented than not yet completely matured techniques such as hydrogen driven processes (Marques et al., 2018; Rogelj et al., 2018). Existing applications running on fossil fuels, such as cars, can indeed relatively easy and cheaply be adapted to run on natural gas instead (Menon and Komarneni, 1998; Choi et al., 2016). Nitrogen is often encountered as an unwanted impurity in natural gas that needs to be removed efficiently before use (Cavenati et al., 2004). On the other hand, post-combustion fuel gas mixtures contain both methane and nitrogen gas among others and the removal of methane of these mixtures is crucial to limit the release of methane into the atmosphere (Shao et al., 2011; Apriliyanto et al., 2018). It is thus clear that the CH_4_/N_2_ is a gas mixture highly relevant for industrial applications.

The aim of this paper is then to study the separating ability of graphene for the CH_4_/N_2_ mixture at room temperature with a focus on the influence of intramolecular force fields in the graphene sheet and the molecular models used for the gas molecules. Section 2 will shortly highlight the computational details of this work, section 3 will be dedicated to the used force fields and the results will be described in section 4. Finally, the conclusions will be presented in section 5.

## 2. Computational Details

We have performed molecular dynamics simulations using DL_POLY v2.2 (Smith et al., 2002) placing a graphene sheet in the middle of a simulation box. We applied periodic boundary conditions in the 3 dimensions allowing sufficient space in the z-direction for assuring that the different copies of the graphene sheet did not interact and to allow gas molecules enough space to escape the graphene sheet. In the x- and y-direction the box size was adapted to a graphene sheet of 840 carbon atoms with an average C-C distance of 1.42 Å, such that there were no defects upon applying the periodic boundary conditions. The box size was thus 51.65 Å × 42.6 Å × 40 Å. All simulations were carried out at 300K in the NVE and the NVT ensembles, using a Hoover thermostat with a relaxation constant of 0.5 ps in the latter case. A cutoff distance of 18 Å was used for all interaction types and a timestep of 1 fs for a simulation time of which 150,000 were for equilibration. Such equilibration time was determined following a well-established procedure previously developed (Faginas-Lago et al., 2017). Convergence was checked by monitoring the time evolution of the energy and temperature. The geometry of gas molecules were optimized at the B3LYP/6-31G** (Hehre et al., 1972; Becke, 1993) level and assumed rigid during the simulations.

## 3. Force Fields

In this work, we used intermolecular potentials based on the ILJ potential (Pirani et al., 2004, 2008) to describe the interactions between different gas molecules on one hand and the interactions between the gas molecules and the graphene sheet on the other (Vekeman et al., 2018a,b). Furthermore, we implemented intramolecular interaction potentials in DL_POLY v2.2. (Smith et al., 2002) to model the flexibility of the sheet. The used force fields are described in detail in the following sections.

### 3.1. Intermolecular Potentials

For the intermolecular force fields, we assume that the interaction can be described by an electrostatic and a non-electrostatic part that are independent from each other

(1) $$\begin{array}{l} \begin{array}{c} V_{\text{tot}}\left(R\right)=V_{\text{nelec}}\left(R\right)+V_{\text{elec}}\left(R\right)\\ \;=V_{\text{ILJ}}\left(R\right)+V_{\text{Coul}}\left(R\right). \end{array} \end{array}$$

For the non-electrostatic part, we used the ILJ potential (Albertí and Lago, 2012, 2013; Faginas-Lago et al., 2016) which has the following form

(2) $$\begin{array}{l} V\left(R\right)=\epsilon \left[\frac{m}{n\left(R\right)-m}{\left(\frac{r_{0}}{R}\right)}^{n\left(R\right)}-\frac{n\left(R\right)}{n\left(R\right)-m}{\left(\frac{r_{0}}{R}\right)}^{m}\right], \end{array}$$

where,

(3) $$\begin{array}{l} n\left(R\right)=\beta +4\left(\frac{R}{r_{0}}\right). \end{array}$$

The potential contains four parameters, one of which, *m*, is fixed depending on the interacting species, in this case, 6, as the species are neutral molecules (Pirani et al., 2008). Furthermore ϵ and *r*_0_ are the well depth and the equilibrium distance, respectively, and have the same meaning as in the standard LJ potential. β is an extra parameter that allow for the tuning of the ILJ potential at long and short interaction distances, where the LJ potential is known to underperform (Albertí et al., 2012; Lago et al., 2013; Faginas-Lago et al., 2015; Faginas Lago, N. et al., 2009). This parameter is loosely related to the hardness of the interacting molecules.

**Figure 1:**
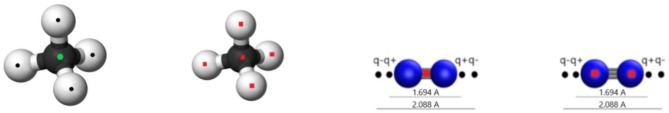
The figure shows the united-atom and atomistic models used in this work for methane and nitrogen. Red squares indicate the location of an ILJ interaction center, black points the location of a charge, and green hexagons an ILJ interaction center and a charge at the same location.

The parameters that were used for the graphene-gas and the gas-gas interactions have been obtained by fitting the ILJ potential, supplemented with a Coulombic sum, to high-level interaction energies at DFT level of the respective systems. The obtained potentials were then benchmarked against DFT and CCSD(T) results as was explained in previous publications (Vekeman et al., 2018a,b). From the results in these articles, here, for both methane and nitrogen, a united-atom and an atomistic model were selected with corresponding partial charges and used for simulations; the used parameters can be found in Table 1. The united-atom model treats the gas molecule as a sphere by putting just one interaction center on the center of mass of the molecule. The atomistic model, on the other hand, puts an interaction center on all atoms of the molecule leading to a higher accuracy, but as well a higher computational cost. For the methane molecule in the united-atom approach, charges calculated by the Hirshfeld population analysis (Hirshfeld, 1977) were used placing a negative charge of –0.148 e on the carbon atom and positive charges of 0.037 e on the hydrogen atoms. For the atomistic approach on the other hand, the best performance was given by not including any charges at all. For the nitrogen molecule, the Cracknell scheme (Cracknell et al., 1996) gave the best performance for both the united-atom and the atomistic approach. In this scheme, both a negative charge of –0.373 e and a positive charge of 0.373 e are positioned on either side of the nitrogen atoms outside of the molecule with the positive charges separated by 1.694 Å and the negative charges by 2.088 Å. The graphene sheet is always represented atomistically by interaction centers on all carbon atoms while electrostatic interactions are neglected. Figurative representations of the used molecular models can be found in Figure 1.

**Table 1:** Interaction parameters for the ILJ potential used in this work to represent the intermolecular potentials in a united-atom or fully atomistic representation.

		**ϵ (kcal mol^**−1**^)**	***r*_0_ (Å)**	**β**
**Methane**			
United-atom	Cm_C_H__4__-Cm_C_H__4__	0.421	4.168	8.215
	C_graph_-Cm_C_H__4__	0.210	3.938	8.185
Atomistic	C_C_H__4__-C_C_H__4__	0.109	3.800	8.027
	C_C_H__4__-H_C_H__4__	0.075	3.628	4.932
	H_C_H__4__-H_C_H__4__	0.005	3.419	4.363
	C_graph_-C_C_H__4__	0.195	3.671	7.745
	C_graph_-H_C_H__4__	0.099	3.727	5.476
**Nitrogen**			
United-atom	Cm_N_2__-Cm_N_2__	0.189	4.322	8.465
	C_graph_-Cm_N_2__	0.123	4.133	6.470
Atomistic	N_N_2__-N_N_2__	0.072	3.902	8.051
	C_graph_-N_N_2__	0.087	3.808	7.861
**Methane/nitrogen mixture**			
United-atom	Cm_C_H__4__-Cm_N_2__	0.288	4.243	7.698
Atomistic	C_C_H__4__-N_N_2__	0.428	3.527	7.923
	H_C_H__4__-N_N_2__	0.002	5.274	6.186

*For methane in the united-atom approach, the Hirshfeld charge scheme was used, whereas for nitrogen in both approaches, the Cracknell scheme was used (Vekeman et al., 2018a,b). Cm referst to the center of mass of the respective molecules*.

A final remark on the used potentials is that, despite the fact that in order to preserve a possible physical interpretation of the β parameters their values must be restricted, we have chosen not to restrict them in order to better describe the potential surfaces determined at the DFT level of theory in previous papers (Vekeman et al., 2018a,b). This same approach has been also used in other works by the ILJ developers (Pacifici et al., 2013; Faginas-Lago et al., 2015, 2016).

### 3.2. Intramolecular Potentials

Intramolecular potentials were introduced in the graphene sheet as mentioned before. In particular, we compared the performance of three different force fields. Firstly we have taken the force field originally developed for carbon nanotubes byWalther et al. (2001)

(4) $$\begin{array}{l} \begin{array}{c} U_{1}\left(r_{ij},\theta_{ijk},\phi_{ijkl}\right)=K_{Cr1}{\left(e^{-\gamma_{1}\left(r_{ij}-r_{C1}\right)}-1\right)}^{2}\\ \;+\frac{1}{2}K_{C\theta 1}{\left(cos\theta_{ijk}-cos\theta_{C1}\right)}^{2}\\ \;+\frac{1}{2}K_{C\phi 1}\left(1-cos\left(2\phi_{ijkl}\right)\right). \end{array} \end{array}$$

This force field contains a stretching, a bending and a torsional term and will be denoted field 1 from now on. The parameters are given as follows: *K*_*Cr*1_ = 114.46 kcal mol^−1^, γ_1_ = 2.1867 Å^−1^, r_*C*1_ = 1.418 Å, *K*_*Cθ*1_ = 134.369 kcal mol^−1^ rad^−2^, θ_*C*1_ = 120° and *K*_*Cϕ*1_ = 6.004 kcal mol^−1^.

Secondly, we took the force field by Kalosakas et al. specifically developed for graphene (Kalosakas et al., 2013)

(5) $$\begin{array}{l} \begin{array}{c} U_{2}\left(r_{ij},\theta_{ijk}\right)=K_{Cr2}{\left(e^{-\gamma_{2}\left(r_{ij}-r_{C2}\right)}-1\right)}^{2}\\ \;+\frac{1}{2}K_{C\theta 2}{\left(cos\theta_{ijk}-\frac{2\pi }{3}\right)}^{2}\\ \;+\frac{1}{2}K_{C\theta 2}^{\prime }{\left(cos\theta_{ijk}-\frac{2\pi }{3}\right)}^{3}, \end{array} \end{array}$$

where the parameters are given as *K*_*Cr*2_ = 131.429 kcal mol^−1^, γ_2_ = 1.960 Å^−1^, r_*C*2_ = 1.420 Å, *K*_*Cθ*2_ = 161.401 kcal mol^−1^ rad^−2^ and $K_{C\theta 2}^{\prime }$ = 92.232 kcal mol^−1^ rad^−3^. This field, denoted field 2 from now on, only takes into account stretching terms and bending terms. However, later on, the same authors extended the field with a torsional term and this constitutes the third field, referred to as field 2 m (field 2 modified) from now on in this work (Fthenakis et al., 2017)

(6) $$\begin{array}{l} \begin{array}{c} U_{3}\left(r_{ij},\theta_{ijk},\phi_{ijkl}\right)=K_{Cr3}{\left(e^{-\gamma_{3}\left(r_{ij}-r_{C3}\right)}-1\right)}^{2}\\ \;+\frac{1}{2}K_{C\theta 3}{\left(cos\theta_{ijk}-\frac{2\pi }{3}\right)}^{2}\\ \;+\frac{1}{2}K_{C\theta 3}^{\prime }{\left(cos\theta_{ijk}-\frac{2\pi }{3}\right)}^{3}\\ \;+\frac{1}{2}K_{C\phi 3}\left(1-cos\left(2\phi_{ijkl}\right)\right). \end{array} \end{array}$$

This field has the same parameters as field 2 with the addition of one extra parameter: *K*_*Cϕ*3_ = 5.304 kcal mol^−1^. Observe that this field 2 m should be considered as the most adequate of the three as it has been specifically developed for graphene and includes the full set of parameters needed to describe the intramolecular motions of the sheet. In fact, it reproduces very accurately the out-of-plane acoustic and optical modes of graphene's phonon dispersion as well as all phonons with frequencies up to 1,000 cm^−1^ (Fthenakis et al., 2017). Anyway, at least in what adsorption concerns, the differences in the results from the three fields are certainly very small, as shown below.

The performance of these three force fields will then be compared to a completely rigid graphene represented as a sheet without intramolecular force field, so explicitly freezing the positions of all carbon atoms. The rigid graphene will be denoted as field 0.

## 4. Results

Our previous work on pure methane and pure nitrogen adsorption on a flexible graphene sheet (Vekeman et al., in press) has clearly shown that graphene has more affinity for the adsorption of methane than for nitrogen and, thus, it can be expected that graphene could serve as a separator for both gases. Furthermore we found that the atomistic model predicted a much stronger methane adsorption than the united-atom model, while for nitrogen these results were quite similar. The united-atom model, on the other hand, showed a lower methane uptake when neglecting the intramolecular movements of the graphene sheet, while there was a smaller discrepancy in these results for the atomistic model.

Since the stronger affinity of graphene for methane than for nitrogen was clearly proven in this previous work, we expect that the graphene sheet could effectively separate this gas mixture. Therefore, in a first set of simulations we have adopted a protocol where we randomly allocated 100 gas molecules (50 CH_4_ + 50 N_2_) over the graphene sheet. The molecules were placed such that at least 5 Å were left in between the different molecules, the edges of the box and the graphene sheet to avoid instantaneous, strongly repulsive interactions at the start of the simulation. We then ran one NVE simulation to allow the system to relax to a physically viable conformation and used the resulting output as input for a first NVT simulation. After this NVT simulation, we kept the molecules that were adsorbed (see below) and deleted the ones that were not. Subsequently, we randomly distributed 100 new gas molecules (50 CH_4_ + 50 N_2_) over the remaining system from the previous simulation to run a new NVT simulation. This protocol was repeated until convergence of the amount of adsorbed molecules. As such, this protocol gives an indication of the amount of molecules that saturates the first adsorption layer and it allows comparison for the different intramolecular force fields and molecular models under study.

As a criterion for adsorption, we considered all molecules closer than 4.6 Å to the graphene sheet to be adsorbed, where the average of the carbon positions in the graphene sheet was used as the zero line. This distance was chosen based on preliminary studies in which the z-density profiles indicated the presence of a first adsorption layer below the distance of 4.6 Å for all studied gases. Similar z-density profiles can be found below where this can be verified.

Table 2 shows the results for the simulations of the adsorption of the methane/nitrogen mixture on the graphene sheet using united-atom models for both gas molecules. The table shows the total amount of molecules that was present and the molar fraction that was adsorbed, methane and nitrogen combined. Then it shows the molar fraction of methane or nitrogen molecules that was present at the start of each respective simulation as X_initial_ and the molar fraction of the respective molecules that was subsequently adsorbed at the end of this simulation as X_adsorbed_.

**Table 2:** Simulation results for the methane/nitrogen mixture using a united-atom approach for the four intramolecular force fields considered in this work.

	**Total**		**CH**_****4****_		**N_**2**_**
**Field**	**Molecules**	**X_**adsorbed**_**	**X_**initial**_**	**X_**adsorbed**_**	**X_**adsorbed**_**
Field 0	100	0.76	0.5	0.86	0.66
	176	0.65	0.53	0.80	0.48
	214	0.63	0.56	0.81	0.39
	236	0.58	0.64	0.68	0.40
	237	0.61	0.64	0.73	0.39
	245	0.65	0.66	0.74	0.47
	259	0.61	0.65	0.74	0.36
	259	0.58	0.68	0.70	0.32
	250	0.57	0.70	0.71	0.25
Field 1	100	0.84	0.50	0.98	0.70
	184	0.70	0.54	0.89	0.47
	228	0.67	0.60	0.85	0.40
	253	0.63	0.66	0.77	0.36
	260	0.63	0.69	0.76	0.33
	264	0.67	0.71	0.81	0.31
Field 2	100	0.84	0.50	0.98	0.70
	184	0.73	0.54	0.90	0.53
	235	0.68	0.60	0.82	0.46
	259	0.65	0.64	0.83	0.33
	268	0.65	0.70	0.78	0.32
	274	0.67	0.72	0.80	0.33
Field 2 m	100	0.89	0.50	1.00	0.78
	189	0.71	0.53	0.89	0.52
	235	0.66	0.60	0.80	0.46
	255	0.65	0.63	0.77	0.44
	266	0.66	0.66	0.83	0.32
	275	0.67	0.71	0.83	0.30

*Results are represented for the total amount of molecules (methane + nitrogen) and the separate methane and nitrogen adsorption within the mixture. X_adsorbed_ indicates the molar faction of the adsorbed molecules*.

Looking at the total amount of molecules that gets adsorbed it is seen that in the initial simulations, most of the molecules are adsorbed (76% for the sheet assumed rigid, 84% for field 1 and field 2 and 89% for field 2 m.), while this fraction lowers in subsequent simulations. In the first simulations, the amount of molecules that is introduced in the system is not enough to fully saturate the graphene sheet and thus most molecules stay will adsorb onto the sheet. When more molecules are gradually introduced, the graphene sheet gets quickly saturated and more molecules will be forced to stay in gas phase in equilibrium with the adsorbed molecules. A closer inspection of the results for methane and nitrogen within the mixture reveals a changing methane/nitrogen ratio from one simulation to the next. Indeed, it can be seen that in the first simulations, a substantial amount of nitrogen molecules is adsorbed on the graphene sheet (66% without intramolecular force field for graphene, 70% with field 1 and field 2 and 78% with field 2 m), while in subsequent simulations, they are steadily removed from the adsorbed layer. The methane molecules are adsorbed preferentially, but in the first simulations there are not enough molecules to completely saturate the graphene sheet allowing nitrogen molecules to adsorb as well. When more methane molecules are added, they occupy more and more space on the graphene surface allowing less space to nitrogen, which is forced to stay in gas phase. In the end, a situation is reached where about 70% of the molecules in the simulation are methane molecules and 30% are nitrogen, consistent over the four graphene sheets. Adding to this, the fraction of methane molecules that adsorbs is much larger than the fraction of nitrogen molecules, giving a first indication that indeed the graphene sheet is well capable of separating methane and nitrogen from each other.

Examining the influence of the flexibility of the graphene sheet on the adsorption, clear differences between the results for the different models are found. First of all, the fraction of the total amount of molecules that is adsorbed if the flexibility of the sheet is not accounted for (about 60%) is lower than when it is explicitly included (about 65%). As methane is dominantly present in the mixture, this result is clearly a reflection of the results for methane within the mixture. While the simulation predicts an adsorption of about 70% of the available methane (note that the initial mole fraction is similar for all fields), the flexible ones increases the prediction to up about 80% of the available methane. For nitrogen, the difference is smaller and anyway with less influence on the total result since the initial fraction is much lower. Keeping in mind that for the pure methane, the united-atom model predicted a lower uptake on the rigid sheet (Vekeman et al., in press) and the dominance of methane in the CH_4_/N_2_ mixture, this results is no surprise. Dreisbach et al. (1999) reported a slowly rising, while converging, methane mole fraction in the methane/nitrogen mixture with a result of 0.733 at 59.8 atm (the highest pressure they measured). In addition, Sudibandriyo et al. (2003) reported a very similar methane mole fraction of 0.732 at the same pressure, while reporting 0.744 at 70 atm. These results coincide very well with our simulations at similar pressures.

The results in Table 3, using the atomistic model for both methane and nitrogen, predict an even more efficient separation. The mole fraction of methane within the mixture reaches values of 0.80 for the rigid model and 0.85 for the flexible ones. The methane is now so strongly adsorbing to the graphene sheet that effectively all nitrogen molecules are forced into gas phase, leaving adsorbed mole fractions close to 0.00 for the flexible representation and 0.07 for the rigid ones. Although methane still dominates the total results, the mole fraction of total molecules that adsorbs is lower, because there is always a portion of nitrogen molecules that never adsorbs. As stated previously, we found in previous work (Vekeman et al., in press) that the methane adsorption predicted by the atomistic model was stronger than for the united-atom model explaining the stronger preference for methane over nitrogen using the atomistic model. This also explains the lower dependence of the adsorption on the flexibility of the graphene sheet as was found for the atomistic pure methane adsorption, as indeed the different intramolecular force fields—or even its complete absence—have little influence in the adsorption when the atomistic model is used.

**Table 3:** Simulation results for the methane/nitrogen mixture using an atomistic approach for the four intramolecular force fields considered in this work.

	**Total**		**CH**_****4****_		**N_**2**_**
**Field**	**Molecules**	**X_**adsorbed**_**	**X_**initial**_**	**X_**adsorbed**_**	**X_**adsorbed**_**
Field 0	100	0.83	0.50	1.00	0.66
	183	0.74	0.55	1.00	0.42
	235	0.73	0.64	1.00	0.26
	272	0.74	0.74	0.97	0.09
	300	0.79	0.81	0.96	0.07
Field 1	100	0.75	0.5	1.00	1.00
	175	0.75	0.57	0.99	0.44
	232	0.72	0.64	0.99	0.23
	267	0.74	0.54	0.97	0.19
	305	0.78	0.79	0.98	0.02
	337	0.78	0.85	0.92	0.00
Field 2	100	0.80	0.50	1.00	0.60
	180	0.72	0.56	0.99	0.38
	229	0.76	0.65	0.98	0.34
	268	0.74	0.73	0.98	0.08
	298	0.78	0.81	0.98	0.00
	333	0.76	0.85	0.89	0.02
Field 2 m	100	0.80	0.50	1.00	0.60
	180	0.69	0.56	0.99	0.33
	225	0.75	0.66	0.99	0.28
	269	0.75	0.74	0.98	0.10
	302	0.79	0.81	0.97	0.02
	338	0.78	0.85	0.92	0.02

*Results are represented for the total amount of molecules (methane + nitrogen) and the separate methane and nitrogen adsorption within the mixture*.

**Figure 2:**
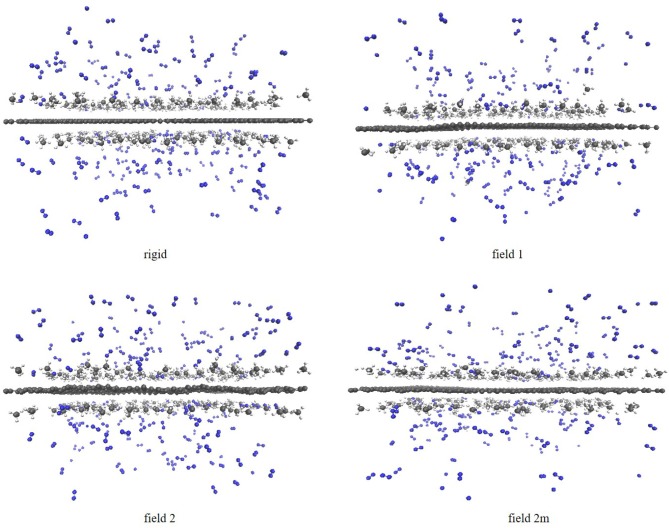
Screenshots of the simulations of the methane/nitrogen mixture on the four graphene sheets using the atomistic model and with 175 methane and 175 nitrogen molecules in the system. From left to right: field 0, field 1, field 2, and field 2 m. Nitorgen atoms are colored in blue, carbon atoms in dark gray and hydrogen atoms in light gray.

Once the saturation point of the graphene sheet was known for the different gas molecules on the different sheets, we were interested to study how the molecules distribute themselves in function of the amount of molecules present in the system. In a second protocol, we therefore ran independent simulations with different, predefined amounts of molecules. More specifically, we ran simulations with 150, 250, and 350 molecules randomly distributed over the graphene sheet, i.e., 75 CH_4_ + 75 N_2_, 125 CH_4_ + 125 N_2_ and 175 CH_4_ + 175 N_2_, respectively. In all cases an NVE simulation was run first to allow the system to relax, after which an NVT simulation was run as a production run. This protocol allows to look at how the adsorption process changes as a function of the amount of molecules introduced into the simulation box. The expected separation of the gases is visible in the screenshots of the simulations with 350 molecules in Figure 2. The methane molecules form a clear adsorption layer on the sheets, while the nitrogen molecules stay in gas phase. While there are some nitrogen molecules entering the adsorption layer and some methane molecules in gas phase, the screenshots once again suggest that the separation is quite effective.

**Figure 3:**
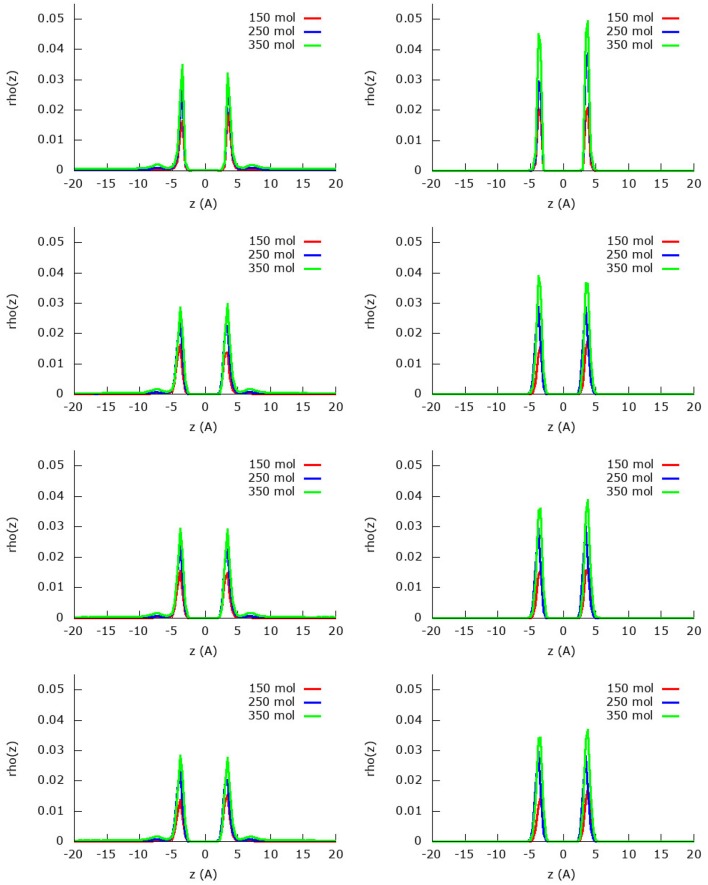
Absolute z-density plots for the four fields considered and using the united-atom **(Left)** or the atomistic **(Right)** models for methane within the methane/nitrogen mixture. From top to bottom field 0, field 1, field 2, and field 2 m.

Figure 3 shows the z-density profiles of the methane adsorption within the methane/nitrogen mixture using a united-atom and an atomistic model for the three different amounts of molecules (150, 250 and 350) of the second protocol. Starting by the united-atom model, it is encountered that—in accordance with the isotherms discussed below and the previously discussed results—when assuming the graphene to be rigid, it adsorbs less methane than the considered flexible sheets. The fact that the adsorption peaks of flexible graphene are lower than those of the rigid one is compensated by a larger broadness, making them of slightly larger area. Indeed, because of the movement of the graphene sheet, the layer will move slightly along, leading to the spread of the molecules along the z-coordinate. After the first very strong adsorption layer, there appears to be a start of a second adsorption layer that arises only in the simulation with 350 molecules. In the two simulations with less molecules (150 and 250 molecules), all methane molecules can be accommodated in the first adsorption layer and no second adsorption layer is observed. For the atomistic model, however, all the methane molecules are adsorbed in the first layer, even for the simulation with 350 molecules. Indeed, it is seen that all 175 methane molecules are adsorbed in the first adsorption layer, no second layer or gas phase being present. Furthermore, the quantities adsorbed by rigid and flexible sheets are almost coincident, with adsorption peaks slightly higher and broader in the case of rigid graphene sheets. Globally, and comparing the two different models, it is once again clear that the atomistic model predicts a larger methane adsorption than the united-atom model.

**Figure 4:**
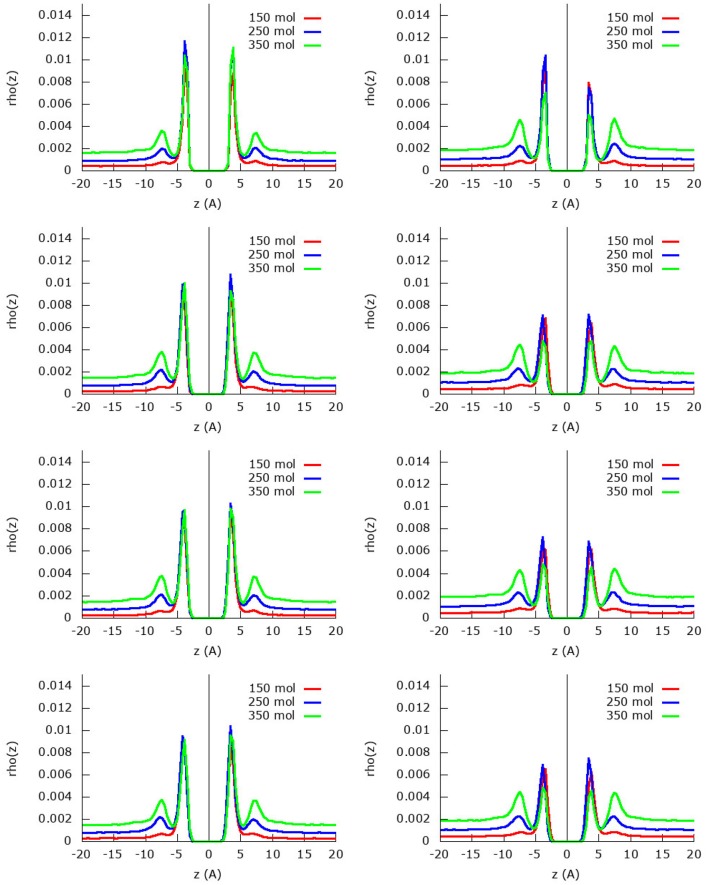
Absolute z-density plots for the four fields considered and using the united-atom **(Left)** or the atomistic **(Right)** models for nitrogen within the methane/nitrogen mixture. From top to bottom field 0, field 1, field 2, and field 2 m.

The z-density profile for the nitrogen molecules from the same simulations are found in Figure 4 for both the united-atom and the atomistic approach. In general, it can be seen that indeed less nitrogen is adsorbed in the first adsorption layer than methane due to the strong competition of the latter. On the other hand, the nitrogen molecules organize themselves in a relatively strong second adsorption layer that is, however, still smaller than the first adsorption layer. Furthermore, there are plenty of nitrogen molecules that are still present in the gas phase as is visible from the area under the curve at distances larger than 10 Å.

For the united-atom model, it is noteworthy to see that the first adsorption layer does not grow upon adding more molecules to the system, the height of the first adsorption peak is as high as in the two subsequent simulations. Whereas the remaining nitrogen molecules stay in gas phase for the simulation with 150 molecules, in the other two simulations (250 and 350 molecules) a strong second adsorption layer is formed. As before, the assumed rigid sheet adsorbs slightly less nitrogen molecules than the flexible sheets.

Looking at the simulations with the atomistic model, the behavior changes quite a bit: the first adsorption layer for nitrogen is smaller for the simulation with the most (350) molecules compared to the other simulations with 150 and 250 methane molecules. This is a consequence of the strong methane adsorption predicted by the atomistic model: the stronger competition allows less nitrogen molecules to enter the first adsorption layer. In the simulation with 150 molecules, the first adsorption layer is not yet completely saturated by methane and nitrogen is allowed space in the first adsorption layer. As the amount of molecules increases, less and less space remains available for nitrogen and the first adsorption peak of nitrogen decreases as a result. Already in the simulation with 150 molecules, there is a small second adsorption layer present, which increases strongly upon increasing the amount of molecules in the system. In the simulation with 350 nitrogen molecules, there are very little nitrogen molecules present in the first adsorption layer, but there is a relatively large second adsorption layer and subsequent gas phase.

**Figure 5:**
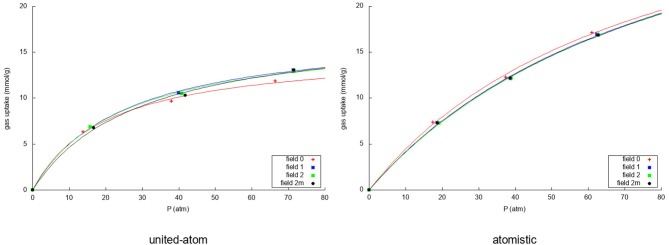
Adsorption isotherms using the united-atom model **(Left)** and the atomistic model **(Right)** for the adsorption over graphene of the methane in the mixture for the four considered models of flexibility of the sheet. In both cases the uptake of methane is plotted against the total pressure of the gas in equilibrium.

**Figure 6:**
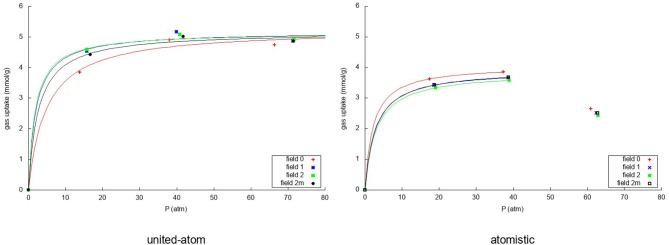
Adsorption isotherms using the united-atom model **(Left)** and the atomistic model **(Right)** for the adsorption over graphene of the nitrogen in the mixture for the four considered models of flexibility of the sheet. In both cases the uptake of nitrogen is plotted against the total pressure of the gas in equilibrium.

Due to the changing widths of the adsorption peaks—due to the internal graphene movement—in Figures 3, 4, it is hard to compare the results for the different fields visually. Instead, visualization is eased by building the adsorption isotherms shown in Figures 5, 6 for methane and nitrogen, respectively. With this aim, the area under the peaks in Figures 3, 4, as well as some equivalent ones determined at different initial number of molecules, were determined. In these figures, the calculated points are plotted together with their fitting to the simple Langmuir equation to give an estimated adsorption isotherm. For the methane molecule, as expected, there is a different behavior for the united-atom and the atomistic models. The former shows clear differences depending on wether the flexibility of graphene is or is not accounted form, the capacity of adsorption increasing with the introduced degree of flexibility in accordance with previous results. The opposite behavior is observed for the atomistic models, although the influence of the explicit inclusion of flexibility is now very small. Once again, the atomistic model predicts a larger uptake of methane molecules than the united-atom model combined with a much slower convergence rate.

For nitrogen, it can be seen that the united-atom model predicts a converged adsorption within the investigated pressure range due to the strong methane competition. The rigid model predicts a slightly lower uptake and slower convergence than the flexible ones. For the atomistic model it is striking that, due to the very strong methane adsorption, the simulation with 350 molecules has very little nitrogen adsorbed on the sheet leading to a very low uptake. For this reason, we have fitted the Langmuir isotherm using only the first two simulation results as it is not intended to describe such a sudden drop in the uptake. Furthermore, the drop is caused by the methane more than by the nitrogen itself. The atomistic model predicts a very slightly lower uptake of nitrogen in the rigid simulation than when considering the flexible representations. As was expected, more nitrogen is adsorbed using the united-atom model than the atomistic model, because the methane is less strongly attracted to the graphene sheet in that case.

Kumar and Rodríguez-Reinoso (2013) have investigated the adsorption of the methane/nitrogen mixture on different carbon-based materials related to graphene: a slit-pore, a carbon nanotube, a carbon foam and a randomized carbon structure. Their grand canonical Monte Carlo simulations were performed in the pressure range between 0 and 50 atm, but with the important difference compared to us that they considered 90/10 and 95/5 methane/nitrogen mixtures. Their methane results were quite similar to ours, especially for the carbon foam, which showed an adsorption of about 14 mmol/g at 50 atm. The adsorption isotherm is, furthermore, very similar in shape to our equivalent atomistic model. The randomized carbon adsorption isotherm has an uptake of 10 mmol/g at 50 atm, while the carbon nanotube and the slit-pore show lower methane uptakes. For the nitrogen molecule, however, their results are somehow different in the sense that they predict the nitrogen uptake to be an order of magnitude lower than that of methane, while in our work the difference is less pronounced. Note that this seemingly big difference can be easily explained by the lower percentages (5 and 10 %) of nitrogen in their mixtures, to be compared with ours (50 %). While this had little influence on the methane molecule, being the dominant adsorbate, it is much more influential on the nitrogen adsorption. Vandenbrande et al. compared different molecular models and experimental results for different MOFs finding the highest methane uptake to be about 18 mmol/g at 70 atm. Moreover, this theoretical result well overestimated the associated experimental number of about 8 mmol/g (Vandenbrande et al., 2017). Similar conclusions are found in the work by Becker et al. where the highest methane adsorption was found to be just above 12 mmol/g (Becker et al., 2017). As such, our results suggest that graphene outperforms many of the investigated MOFs for which adsorption results have been reported in the literature as was also indicated by Kumar et al. (2013) and Wu et al. (2015).

Finally, we have calculated the selectivity as follows

(7) $$\begin{array}{l} S_{\text{AB}}=\frac{{\left(\frac{x_{\text{A}}}{x_{\text{B}}}\right)}_{\text{adsorbed}}}{{\left(\frac{y_{A}}{y_{B}}\right)}_{\text{bulk}}}, \end{array}$$

where, *x* is the mole fraction adsorbed of the specified molecule, while *y* is the mole fraction in the bulk of the specified molecule.

**Figure 7:**
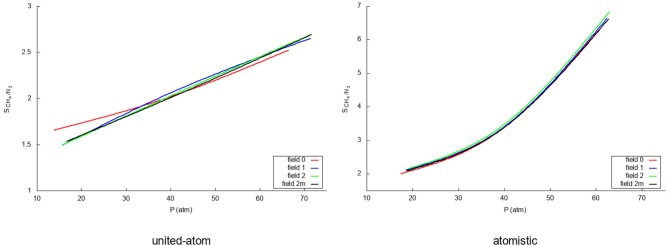
Selectivities for the methane/nitrogen mixture for the four different fields using a united-atom **(Left)** and atomistic **(Right)** model for the gas molecules.

Figure 7 shows how the separation capacity of graphene for the methane/nitrogen mixture varies with pressure depending both on the way in which the flexibility of graphene is considered and on the used intermolecular model. For the united-atom molecule, the rigid representation behaves again somewhat different than the flexible ones. Whereas the flexible models show a linear rise of the selectivity within the pressure range that was investigated, the rigid simulation shows a slightly curved increase which crosses the flexible curves at around 35 atm. Within the pressure range considered here, the deviation is more evident at low pressures and, indeed, the overestimation produced by completely neglecting the intramolecular motion of graphene is above 10%. Contrarily, the atomistic model, shows little influence of the flexibility with an exponential increase with rising pressure. As was seen previously, the stronger attraction of the atomistic methane strongly favors the methane adsorption leading to an even higher selectivity. An important point to note is the fact that completely neglecting the atomic structure of the gases leads to a clear underestimation of the selectivity of graphene. In fact, even though the effect is not so exaggerated at low pressures, at higher pressures the selectivity predicted by the oversimplified united-atom model is sensibly less than half the determined by the atomistic model. At any rate, the selectivity is good in all cases and rises with rising pressure since more methane molecules enter the system, pushing the nitrogen molecules out of the adsorption layer.

Even though (Dreisbach et al., 1999) have not explicitly reported selectivities for their carbon pores, the methane uptake is about five times higher at 60 atm than the nitrogen uptake as was the case in the work of methane/nitrogen adsorption on wet Tiffany coals by Fitzgerald et al. (2005). Both results are in agreement with the selectivities from our atomistic models in this work, which represents another argument justifying the superiority of such model over the simple united-atom one. In particular, it is noteworthy to stress that the latter produces values of the selectivity that are approximately half of those predicted by the atomistic model. Moreover, it is also significant the deviation-especially at low pressures-of the rigid graphene model with respect to the flexible ones when using the united-atom approach to describe the gas molecules.

## 5. Conclusions

In this work, we have studied the influence of the flexibility of the graphene sheet on its separation ability of the methane/nitrogen gas mixture. The flexibility was introduced via three intramolecular force fields taken from the literature, two of which contained stretching, bonding and torsional terms, while a third one lacked the latter contribution. Two fields were specifically designed for graphene, while a third one was originally intended for use on carbon nanotubes. Furthermore we studied the different behavior of a united-atom model and an atomistic model during these simulations.

In general, we have confirmed that graphene shows a strong preference for methane over nitrogen. Using both models, the methane posed a strong competition toward the nitrogen and pushed the nitrogen out of the first adsorption layer, forcing it to form a second adsorption layer or go into gas phase. This effect was found stronger for the atomistic gas models than for the united-atom models, which is expected to give a poorer representation as it does not takes into account orientation effects. The difference, which is at times large, calls for care when assuming united-atom models in these types of simulations.

Concerning the treatment of flexibility, we showed that, indeed, it influences the behavior of the graphene sheet as a separator of the methane/nitrogen gas mixture. For the united-atom model, neglecting the flexibility of the graphene sheet leads to predict lower methane uptakes that if such flexibility is taken into account. The atomistic model on the other hand predicted a slightly higher methane uptake. Similar results were found for nitrogen, leading to the conclusion that for the united-atom model, a lower amount of gas molecules in general is predicted to be adsorbed if the sheet is supposed to be rigid. The atomistic model, on the other hand, predicts a larger general gas adsorption also on the considered rigid graphene sheet. Observing the combination of these results in the selectivity of the four models of the intramolecular motions of graphene, we find that the united-atom model in conjunction with the rigid representation predicts the lowest selectivity for the methane/nitrogen mixture in the largest portion of the pressure range investigated, while the atomistic model shows very similar behavior for the rigid and the flexible graphene models. Moreover, since graphene is, in fact, flexible, it is then clear that in order to safely disregard its internal movements an atomistic model is definitely required, the simplified united-atom model being absolutely inadequate. On the other hand, if the flexibility of graphene is explicitly accounted for by means of the appropriate force field -the field 2 m (Fthenakis et al., 2017) conceptually being the one of choice-, both atomistic and united-atom models provide very similar results.

## Author Contributions

JV, IG, and AS ran the first principles calculations and developed the subsequent interaction force field. NF-L, AL, and MR developed and ran the theoretical and molecular dynamics calculations.

### Conflict of Interest Statement

The authors declare that the research was conducted in the absence of any commercial or financial relationships that could be construed as a potential conflict of interest.
